# Kinetic Measurements Reveal Enhanced Protein-Protein Interactions at Intercellular Junctions

**DOI:** 10.1038/srep23623

**Published:** 2016-03-24

**Authors:** Nitesh Shashikanth, Meridith A. Kisting, Deborah E. Leckband

**Affiliations:** 1 Department of Biochemistry, University of Illinois Urbana-Champaign, Urbana, Illinois- 61801, USA; 2 Department of Chemical and Biomolecular Engineering, and University of Illinois Urbana-Champaign, Urbana, Illinois- 61801, USA; 3 Department of Chemistry, University of Illinois Urbana-Champaign, Urbana, Illinois- 61801, USA

## Abstract

The binding properties of adhesion proteins are typically quantified from measurements with soluble fragments, under conditions that differ radically from the confined microenvironment of membrane bound proteins in adhesion zones. Using classical cadherin as a model adhesion protein, we tested the postulate that confinement within quasi two-dimensional intercellular gaps exposes weak protein interactions that are not detected in solution binding assays. Micropipette-based measurements of cadherin-mediated, cell-cell binding kinetics identified a unique kinetic signature that reflects both adhesive (*trans*) bonds between cadherins on opposing cells and lateral (*cis*) interactions between cadherins on the same cell. In solution, proposed lateral interactions were not detected, even at high cadherin concentrations. Mutations postulated to disrupt lateral cadherin association altered the kinetic signatures, but did not affect the adhesive (*trans*) binding affinity. Perturbed kinetics further coincided with altered cadherin distributions at junctions, wound healing dynamics, and paracellular permeability. Intercellular binding kinetics thus revealed cadherin interactions that occur within confined, intermembrane gaps but not in solution. Findings further demonstrate the impact of these revealed interactions on the organization and function of intercellular junctions.

Adhesion proteins function in confined regions between cell membranes and extracellular matrix or adjacent cells. Yet binding mechanisms of adhesion proteins, associated affinities, and adhesion energies are typically based on investigations of binding between soluble, recombinant fragments of membrane proteins. Apart from possible functional perturbations stemming from the use of recombinant fragments, theoretical models and experimental findings indicate that affinities measured in solution differ quantitatively from those determined in quasi two-dimensional gaps at adhesive contacts^1^,^2^,^3^,^4^,^5^,^6^,^7^,^8^,^9^,^10^. The physical and chemical constraints within adhesion zones are also predicted to alter molecular mechanism(s) driving protein organization^8^.

Cadherins are exemplary proteins for testing whether confinement alters adhesion protein interactions. They are transmembrane cell-to-cell adhesion proteins that form homophilic bonds between extracellular domains of cadherins on adjacent cells. They can assemble into dense plaques at intercellular adhesions that are characterized by high cadherin densities associated with a dense actin belt^11^,^12^. Intermembrane gaps at cadherin-mediated adhesion zones are typically ~35 nm^13^,^14^. Crystal structures and solution binding studies of recombinant extracellular fragments suggested that cadherins assembled adhesive junctions mainly by forming adhesive bonds between the extracellular domains of opposing proteins, which we refer to as ‘*trans* bonds’ or ‘strand-swapped dimers’ (Fig. 1a)^15^. However, molecular force measurements quantified binding between cadherin extracellular domains, in configurations analogous to proteins at intercellular gaps. Force measurements identified multiple cadherin interactions that involved different sub regions of the extracellular region, although it was unclear whether those interactions reflected *trans* or *cis* bonds (Reviewed in^16^). Although there was some biophysical evidence suggesting that lateral ectodomain dimerization enhanced activity, diverse biophysical approaches did not detect lateral bonds between soluble, recombinant ectodomains^17^,^18^,^19^,^20^,^21^. In addition to the initial ‘strand-swapped dimer’ (Fig. 1b), structural analyses identified two, additional possible binding interfaces: one *trans* bond between opposed proteins, deemed the ‘X-dimer’ (Fig. 1a), and a potential *cis* (lateral) binding interface (Fig. 1c)^22^,^23^. Although X-dimers (Fig. 1a) form in solution, the lateral interaction observed in crystal structures was not detected in solution, even at high protein concentrations^23^,^24^,^25^. However, mutations at the putative *cis* (lateral) binding interface disrupted cadherin organization at artificial junctions between giant vesicles^13^ and perturbed cadherin distributions at cell-cell junctions^26^.

Molecular Dynamics and Monte Carlo simulations suggested a possible physical basis for apparent discrepancies between solution binding data, crystal structures, and force probe measurements. The simulations predicted that binding between flexible cadherin ectodomains on opposing cells pins the proteins, and reduces their configurational entropy. This could in turn reduce the entropic repulsion between proteins on the same membrane, enabling the formation of weak lateral (*cis*) protein-protein bonds. Confining adhering cadherins within intermembrane adhesion zones could thus expose lateral interactions that are otherwise not observed^7^,^8^.

The simulation results further suggested that cadherins on the same membrane associate laterally (*cis* bonds) in adhesion zones, but that *cis* bond formation requires the initial pinning of opposing, adhering cadherin ectodomains. However, cadherins form clusters of >5 proteins on free (unconfined) membranes^14^, and cadherin connections to actin, actin assembly/disassembly dynamics, endocytosis, and cadherin binding interactions reportedly regulate the assembly of much larger cadherin clusters at intercellular contacts^27^,^28^,^29^,^30^. It is therefore still an open question as to the quantitative effects of confinement on cadherin interactions. A further open question is whether predicted confinement-enhanced cadherin interactions alter physiologically relevant properties of intercellular junctions.

Results presented in this study of E-cadherin mediated, intercellular binding kinetics, provide quantitative evidence for confinement-enabled cadherin interactions within intercellular junctions. Measurements of the adhesion frequency between cell pairs revealed a unique kinetic signature that reflects initial *trans* (adhesive) cadherin bond formation, which is then followed by a second kinetic process attributable to the onset of *cis* (lateral) interactions within tens of seconds of initial cell-cell contact. Kinetic studies and super resolution imaging of cadherin mutants that were postulated to disrupt lateral interactions, support this interpretation. Measurements with recombinant cadherin ectodomains and with full-length cadherins further confirmed that the kinetic signatures were independent of cytoskeletal interactions. These results thus provide biophysical evidence that quasi two-dimensional confinement quantitatively alters adhesion protein (cadherin) interactions, which affect the assembly and function of intercellular junctions. Comparisons of kinetics with cell and tissue functions reveal the potential physiological significance of these findings.

## Materials and Methods

### Plasmids, cell lines and proteins

CEP 4.1 plasmids encoding the hexahistidine-tagged, wild type mouse E-cadherin ectodomain (E-Cad-His_6_ in this paper) and mutants V81D and L175D and the double mutant V81D/L175D were from Dr. Lawrence Shapiro (Columbia University, NY)^13^. The plasmid encoding the full-length Human E-cadherin-GFP (Hu-E-Cad-GFP) was from Dr. Jennifer Stow (Addgene plasmid #28009)^31^. V81D and V175D mutations to the Hu-E-Cad-GFP construct were introduced, using the Stratgene site-directed mutagenesis kit.

The human Embryonic Kidney (HEK) 293T cell line was from the American Type Culture Collection (Manassas, VA). A431D Epidermoid Carcinoma cells, which do not express endogenous E-cadherin^32^ was from Dr. Keith Johnson (University of Nebraska, Lincoln). Cells were cultured in Dulbecco’s Minimum Eagle Medium (DMEM) containing 10% fetal bovine serum (FBS) (Life Technologies, Carlsbad, CA) in a 5% CO_2_ atmosphere at 37 °C. Cell lines that stably expressed the soluble proteins were generated, by transfecting Human Embryonic Kidney 293T cells with the constructs indicated above, using Lipofectamine 2000 (Invitrogen, Grand Island, NY) according to the manufacturer’s instructions.

HEK 293T lines stably expressing hexahistidine-tagged, soluble ectodomains were selected with 200 μg/mL Hygromycin B (Invitrogen). Western blots of the culture media confirmed protein expression by individual colonies. The colonies that expressed the highest levels of soluble protein were chosen for further soluble protein production. Secreted, hexahistidine-tagged proteins were purified from filtered culture medium, by Ni/NTA affinity chromatography, and subsequent ion-exchange chromatography.

Stable cell lines that express full-length Hu-E-Cad-GFP or its mutants were generated by transfecting A431D cells, using Lipofectamine 2000, and selection using geneticin sulfate (400 μg/mL). Fluorescence Activated Cell Sorting (FACS) was used to select cell populations with similar median GFP intensity, indicating similar cadherin surface expression (cadherins/μm^2^).

### Red Blood Cell (RBC) isolation, modification, and E-cadherin labeling

The surfaces of the Red Blood Cells (RBCs) used in adhesion frequency measurements, were ectopically modified with oriented E-cadherin extracellular domains with C-terminal His_6_ tags^33^ (Fig. 2b). The RBCs were isolated from human whole blood collected from healthy adult subjects, by informed consent, according to protocol #08669, which was approved by the Institutional Review Board (IRB) of the University of Illinois at Urbana-Champaign, in accordance with the Institutional Review Board guidelines for use of human subjects. The IRB approved protocol (#08669) includes experimental methods for the isolation and use of RBCs. The erythrocyte surfaces were modified with mouse anti-hexahistidine monoclonal antibody (Aviva Systems Biology, San Diego, CA), as described^34^. The surface density of antibody (antibodies/μm^2^) on the RBC surface was quantified by flow cytometry, as described below, using a goat anti-mouse-CFL 647 IgG (Santa Cruz Biotech, TX). Cells were then modified with oriented, E-Cad-His_6_ ectodomains, by incubation with 5 μg/mL E-Cad-His_6_ in 1X Phosphate Buffered Saline (PBS), containing 1w/v% Bovine Serum Albumin (BSA) and 2mM CaCl_2,_ for 45 min, with gentle agitation at 4 °C.

### Quantification of cadherin surface expression

Flow cytometry measurements quantified the density of surface-expressed cadherin (cadherins/μm^2^). E-cadherin expressing cells were labeled with the primary, anti-E-cadherin antibody DECMA-1 (Sigma-Aldrich, St. Louis, MO), which binds the fifth extracellular repeat domain (EC5) of murine and human E-cadherin^35^. The secondary antibody was CFL-647 -conjugated anti-rat IgG (whole antibody, Santa Cruz Biotech, TX). The antibody labeling was done in 1X PBS containing 1 w/v% BSA at pH 7.4. The fluorescence intensities of labeled cells were measured with an LSR II flow cytometer (BD Biosciences). The calibration curve used to relate the fluorescence intensity to the cadherin surface density was generated with calibrated Alexa 647-labeled standard beads (Bangs Laboratories, Fishers, IN).

### Micropipette Measurements Of Cell Binding Kinetics

Adhesion frequency measurements quantified the intercellular binding probability as a function of contact time, by using opposing micropipettes to manipulate interacting cell pairs (Fig. 2)^2^. The measured binding probability P(t) is the ratio of the number of binding events n_b_ to the total N_T_ cell-cell touches, n_b_/N_T_, and is a function of the number of cell-to-cell bonds^2^. In these measurements, a cadherin-expressing cell and a RBC with surface-bound, His-tagged cadherin ectodomains were partially aspirated into opposing glass micropipettes (Fig. 2a,b). The experimental chamber contained L15 medium (Invitrogen, Carlsbad, CA) supplemented with 1 w/v% BSA and 2mM CaCl_2_, and diluted 1:1 with deionized water. This hypo-osmotic solution keeps the RBCs rounded. Large differences in ionic strength have a slight effect on N-cadherin affinity^36^, but halving the salt concentration, as in our case, would only increase the Debye length from ~0.8 nm to ~1.2 nm^37^. However, because the affinities of WT and mutant E-cadherin were measured under identical conditions, the trends should be the same at the given osmolarity. Cells were observed with a 100× oil immersion objective on a Zeiss Axiovert 200 microscope, and images were recorded with a Manta G201B camera (AVT technologies) interfaced with a high resolution (1080 × 720 pixels), flat screen monitor. The contact time was manipulated with computer-controlled, piezo-electric manipulators programmed to repeatedly bring the two cells into contact for defined intervals. The visualized contact area was controlled at 6 ± 1 μm^2^ during a single set of measurements. Binding events were identified from surface deformations of the RBCs during cell separation and recoil at bond rupture. Each cell pair was tested for 50 repetitive cell-cell touches (N_T_ = 50), and each contact time in the figure represents measurements with at least three different cell pairs. The mean and standard deviation of each set of 50 tests was determined from the Bernoulli distribution^2^,^34^. The probabilities P shown in the graphs are the average of measurements with 3 cell pairs, and the error bars indicate the standard errors from the mean of the 3 sets of measurements with different cell pairs, at each time point.

### Scratch Wound Healing Assay

Wound healing assays were performed with A431D cells that stably expressed WT Hu-E-Cad-GFP or its V81D, V175D, or V81D/V175D mutants. Cells were grown to confluence in glass bottom dishes (Cell E & G, Houston, TX). After wounding the monolayer with a 200 μL pipet tip, the cells were washed gently with medium three times, and imaged at the same spot at approximately 2 hour time intervals, using a 20X objective (phase contrast) on a Zeiss Axiovert 200 microscope. In the intervening times, the cells were maintained at 37 °C in 5% CO_2_. These images were analyzed using the Zeiss Axiovision (Version 4.2) software. For each image, outlines of the gaps were drawn on both the leading edges over a length of 150 μm, and the remaining gap area was calculated (N = 25). The percentage of the remaining gap area, relative to the initial wound area, was plotted as a function of incubation time for the different cell lines. Student t-tests were performed to test for significance from the standard errors for all cell lines at the 4 hour time point.

### Paracellular Permeability

A431D cells were grown to confluence on Corning Transwell inserts for 5–7 days to ensure that cells covered the entire surface of the insert. On the day of the assay, the cells were washed with Hank’s Balanced Buffer Solution (HBSS), and the upper chamber was filled with HBSS that contained a defined concentration of BSA conjugated to Evans blue (8:1 ratio, referred to as ‘stock’). After 2.5 hours, samples from the lower chamber were collected in triplicate for each experiment. The dye fluorescence was measured using a SpectraMax® M2 Multi-detection spectrophotometer, with excitation at 540 nm and emission at 680 nm. A calibration curve was plotted for each experiment, using defined concentrations of Evans Blue-BSA. The percentage of dye leaked into the lower chamber was determined from the sample mean fluorescence intensities, calculated from the slope of the calibration curve.

### Super Resolution- Structured Illumination Microscopy (SR-SIM)

A431D cells expressing full length, GFP-tagged E-cadherin or its mutants were cultured in 35mm glass bottom dishes. On the day of the experiment, the cells were nearly 80% confluent. These cells were washed 3 times with DMEM (without phenol red) and immediately imaged for GFP by excitation with a 488nm laser on a Zeiss ELYRA 700 microscope with a 64X-oil immersion lens. The images were processed using the Structured Illumination module of the Zeiss (Zen 2011) software to obtain the super-resolved images of Hu-E-Cad-GFP. The spatial resolution of the instrument is 150 nm.

## Results

### Distinct Kinetic Signature Requires Putative Lateral (Cis) Cadherin Interactions

**Figure 1:**
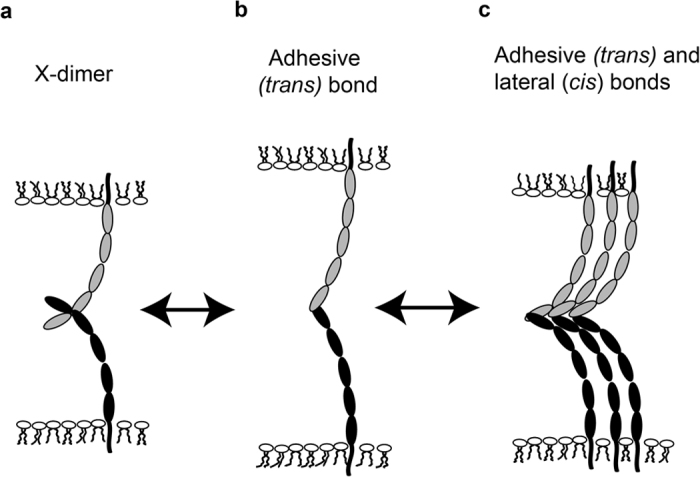
Proposed Cadherin Interactions at Cell-Cell Contacts. **(a)** Cadherins from opposing cells interact to form a transient intermediate, X-dimer, **(b)** which then converts to a an adhesive (*trans*) bond. **(c)** Upon adhesive (*trans*) bond formation, reduced protein fluctuations would result in decreased repulsion between adjacent proteins, and enable the formation of lateral interactions and larger cadherin clusters.

**Figure 2:**
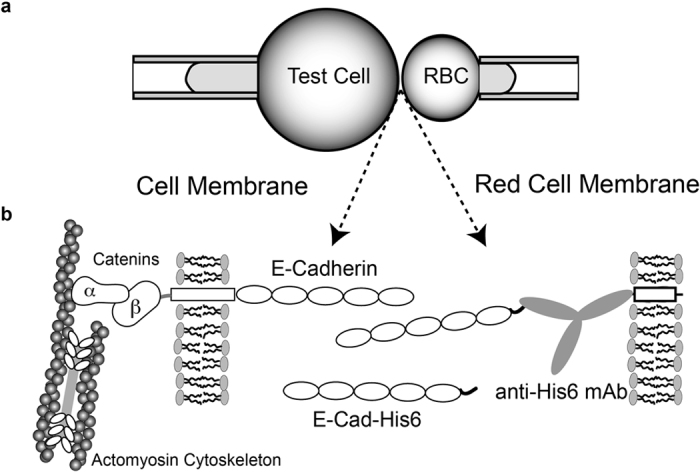
Illustration of the Micropipette Setup. a) A test cell (for example, A431D cells) expressing full-length cadherin is aspirated into the left micropipette, and a Red Blood Cell (RBC) ectopically modified with E-Cad-His_6_ is aspirated into the right micropipette b) Protein orientations on the opposing test cell and the RBC. The RBC is covalently modified with anti-His6 antibodies, which capture and orient the histidine tagged E-Cadherin extracelluar domains. The cells are repetitively brought into contact for a defined period (and contact area) and retracted with piezoelectric manipulators. Adhesion events are quantified from visible RBC deformations and recoil upon bond failure. In the left micropipette, cells can be replaced with a modified RBC, as in the right pipette, in order to quantify binding between ectodomains only.

**Figure 3:**
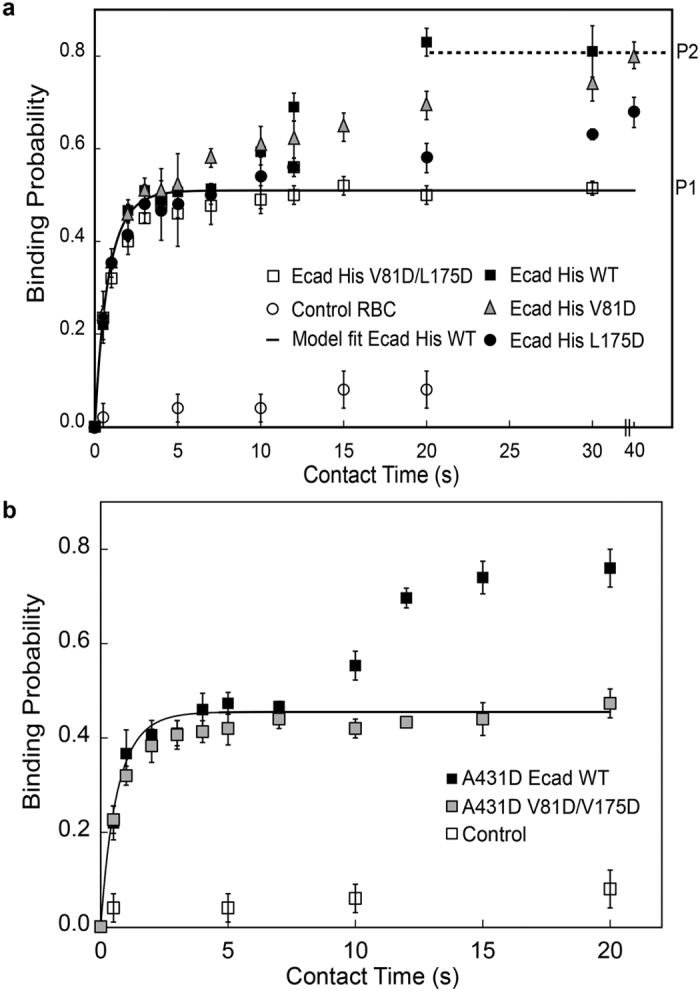
Binding Kinetics of Soluble and Membrane Bound E-Cadherin Variants. **(a)** Mutations at the putative lateral binding interface alter the binding kinetics of recombinant E-cadherin ectodomains. The plot shows the binding probability versus contact time between two RBCs ectopically-modified with WT mouse E-Cad-His_6_ (black squares) or the mutants L175D (black circles), V81D (gray triangles) and V81D/L175D (white squares). The cadherin surface densities are summarized in Table 1. The solid line is a nonlinear least squares fit of Eq. 1 to the data corresponding to the first kinetic (WT mouse E-cadherin), with best fit parameters summarized in Table 1. Controls (white circles) used RBCs without immobilized E-Cad-His_6_. **(b)** Binding probability versus contact time between RBCs ectopically-modified with mouse E-Cad-His_6_ (WT or Double mutant), and A431D cells expressing Hu-E-Cad-GFP WT (black squares) or the V81D/L175D mutant (grey squares). The cadherin densities on the cell surface are summarized in Table 1. The solid line is the nonlinear least squares fit to Eq. 1 of data corresponding to the first kinetic step (Hu-E-Cad-GFP WT), with best fit parameters given in the text and summarized in Table 1. Controls (white squares) used A431D E-Cad-GFP WT cells and RBCs without immobilized E-Cad-His_6_.

Cadherin-mediated, intercellular adhesion frequency measurements, using the dual micropipette set-up (Fig. 2a), were first carried out with RBCs ectopically modified with mouse E-Cad-His_6_, in both micropipettes. As shown in Fig 3a, the binding probability initially rose to an initial steady-state plateau P1 ~ 0.5, followed by a brief lag, and then a second increase in binding probability to a plateau at P2 ~ 0.8. This distinct two-stage or ‘biphasic’ kinetic signature is a general feature of all type I classical cadherins studied so far^34^,^38^,^39^,^40^. Prior studies with binding site mutants and domain deletion mutants demonstrated that the initial increase to the first plateau, P1 is due to *trans* (adhesive) binding (Fig. 1b)^38^,^40^.

In measurements with the V81D and L175D mutants, the initial rise in binding probability was similar to that of WT mouse E-Cad-His_6_, at comparable surface densities. However, the further increase in binding probability (second stage) was much slower (Fig. 3a). With the V81D mutant, at 40s, the second rise reached a probability of P2 ~ 0.8, similar to that of WT E-Cad-His_6_. With L175D, the second rise was even slower, and the binding probability at 40 sec (~0.7) was slightly lower than that of WT E-cadherin (p = 0.05, N_exp_ = 3). The final steady state plateau could not be determined for L175D, on account of the instrument drift over much longer contact times. The double mutant V81D/L175D completely abolished the second increase in binding probability (to P2). Controls with RBCs modified only with anti-hexahistidine antibody confirmed the cadherin-specificity of the measured adhesion frequency time courses (Fig. 3a).

The initial binding step due to *trans* (adhesive) bond formation can be described by a simple receptor (R) -ligand (L) binding reaction^2^,^34^,^38^,


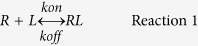


where the binding and dissociation rates are *k*_*on*_ and *k*_*off*_, respectively. The analytical expression for the time-dependent, binding probability P(t) for the above reaction is^2^:


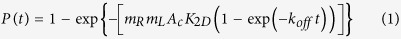


Here, m_L_ and m_R_ are the receptor and ligand surface densities (cadherins/μm^2^) on the two cells, A_c_ is the contact area (μm^2^), K_2D_ is the two-dimensional binding affinity (μm^2^), and k_off_ is the dissociation rate (s^−1^). The values of the ligand surface densities and contact areas (cadherins/μm^2^) were known. Thus, the two-dimensional affinity K_2D_ and off rate k_off_ for *trans*-binding were estimated from fits of Equation 1 to the data corresponding to the first binding step—that is, the rise to P1^33^,^34^,^38^,^40^.

In order to quantify the *trans* (adhesive) binding parameters, we first parsed the kinetic data between the initial rise to P1, and the subsequent rise to P2 (see Fig. 3a), using a non-linear lack-of-fit F-test^34^. The F-test compares the least squares residuals of data fits to the model (Eq. 1) with the intrinsic variability in the data. If the test statistic exceeded the critical value for a given time point, then the time point did not fit the *trans* binding model, and was attributed to the second kinetic step. In this study, *trans* binding, as modeled by Eq. 1, described the rise to P1, but not the subsequent increase to P2 at t >~10 sec. The dissociation rate and two-dimensional affinity of *trans* (adhesive bonds) were determined from fits of Eq. 1 to the maximum number of points in each data set that did not fail the lack-of-fit test. In the case of the V81D/L175D mutant, all the data points were included in the fits. The lines in Fig. 3 are the weighted, nonlinear least squares fits (OriginLab 9.0, Northampton, MA) of data corresponding to the initial rise to P1, with the best-fit parameters summarized in Table 1.

The data fits revealed that the 2D-binding affinity (K_2D_) and off-rate (k_off_) for *trans* binding (Fig. 1a) by WT mouse E-Cadherin and both mutants (see Table 1) were statistically similar. The p-values at the 95% confidence level in two-tailed statistical analyses were >0.1, regardless of the pairwise comparisons. Although the *cis* interface mutations altered the kinetic profiles qualitatively, they did not affect the *trans* binding parameters, in agreement with solution binding measurements of recombinant ectodomains with these same mutations^13^.

### Two-Stage Kinetic Signatures Are Independent Of The Cadherin Cytoplasmic Domain

To test whether the kinetic behavior measured with cadherin ectodomains reflected the behavior of membrane-bound cadherins, we conducted measurements with A431D cells that stably expressed human E-cadherin-GFP or its *cis* (lateral) interface mutants. Fig. 3b shows the adhesion frequency time courses measured between A431D cells expressing either WT Hu-E-Cad-GFP or the double mutant V81D/V175D Hu-E-Cad-GFP and RBCs ectopically labeled with the same ectodomain—that is, with WT E-Cad or the V81D/V175D mutant. Based on quantitative flow cytometry measurements, the surface expression of Hu-E-Cad-GFP (WT or V81D/L175D) on A431D cells were similar (Table 1). The kinetics were also qualitatively and quantitatively similar to those measured with the ectodomains alone (compare with Fig. 3a). Binding between cells expressing WT Hu-E-Cad-GFP and RBCs modified with WT E-Cad-His_6_ exhibited a two-stage kinetic time course, in which the second rise to P2 occurred after 7 sec. In measurements between cells expressing V81D/V175D Hu-E-Cad-GFP and RBCs modified with V81D/V175D E-Cad-His_6_, the second increase in binding probability was abolished, but the determined *trans* (adhesive) binding affinities and off-rates were statistically similar to WT E-cadherin (Table 1, p >0.1). Thus, within the initial 40s of cell-cell contact, the quantitative and qualitative features of the E-cadherin mediated binding kinetics were independent of the cytoplasmic domain.

### Lateral (Cis)-Interface Mutants Alter Cadherin Organization At Junctions But Not On Free Membranes

Super Resolution Structured Illumination Microscopy (SR-SIM) imaging then determined whether the putative *cis* binding-interface between ectodomains altered the organization of Hu-E-cadherin both at A431D intercellular junctions and at the free edges of cells outside contact regions. Different from super resolution Stochastic Optical Reconstruction Microscopy (STORM) imaging of Hu-E-cadherin nanoclusters^14^, SR-SIM imaged cadherin clusters on live cells without fixation, antibody labeling or use of oxygen-scavenging buffers to enhance the image stability. The SR SIM resolution (~150 nm) is less than STORM but twice that of confocal microscopy, and sufficient to characterize cadherin clusters with diameters >20 nm^33^.

**Figure 4:**
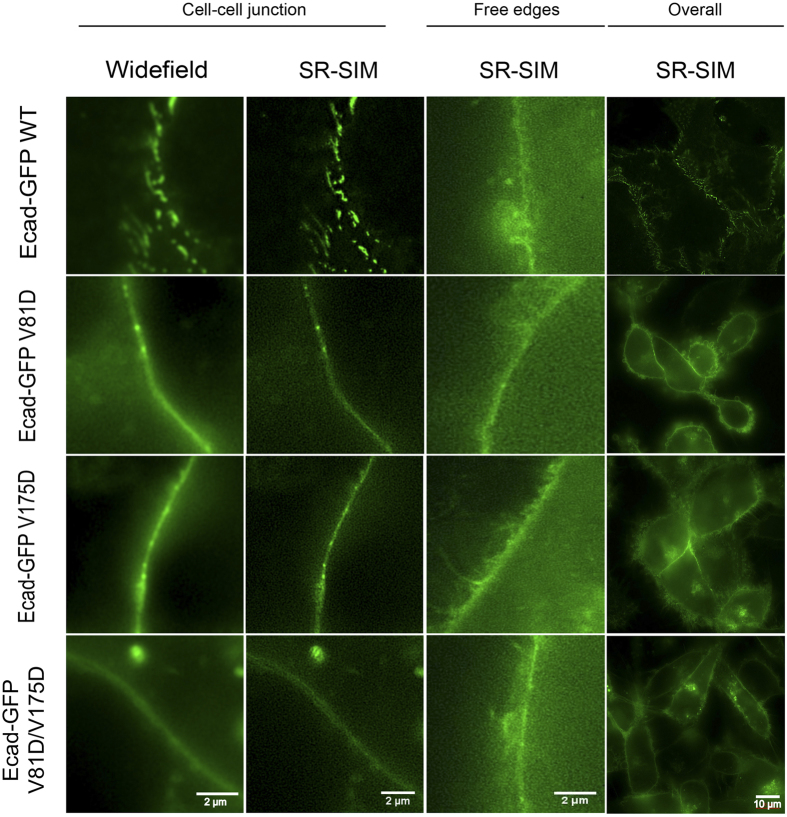
Structured Illumination Microscopy (SIM) images reveal differences in cadherin organization among mutants. SR-SIM (Super Resolution- Structured Illumination Microscopy) of live A431D cells expressing the different Hu-E-Cad-GFP variants. The images compare wide field and processed SR-SIM images at a resolution of 150 nm. Images are shown for A431D cells expressing the different E-Cadherin variants, at cell-cell junctions. From left to right, the first two columns compare organization of E-cadherin GFP at cell-cell contacts, as observed by widefield or SR-SIM. The third column shows E-cadherin localization at contact-free edges of the cells. The fourth column shows the organization of E-Cad-GFP in a group of cells. Scale bars are 10 μm (4^th^ column) and 2 μm for the remaining images. The images shown are from different cells from different replicate experiments, and are not a subset of the zoomed out images of the 4^th^ column.

SR SIM images revealed distinct punctate cadherin clusters at junctions between A431D cells stably expressing WT Hu-E-Cad-GFP. The adhesion plaques observed between live (unfixed) A431D cells expressing WT Hu-E-Cad-GFP (Fig. 4) were similar to the large, dense cadherin plaques at junctions observed in 3D-STORM images^14^. However, such defined, puncta were not visible with any of the *cis*-interface mutants, and clusters were smaller and more diffuse at cell-cell junctions (Fig. 4). Outside of cell-cell contacts, there was no apparent cadherin organization, and the E-Cad-GFP was diffuse, even on cells expressing WT Hu-E-Cad-GFP (Fig. 4). Thus, the *cis* interface mutants altered the organization of cadherins at cell-cell junctions, but did not affect cadherin organization outside of confined adhesion zones.

### Perturbed Cadherin Interactions Alter Intercellular Junction Integrity

**Figure 5:**
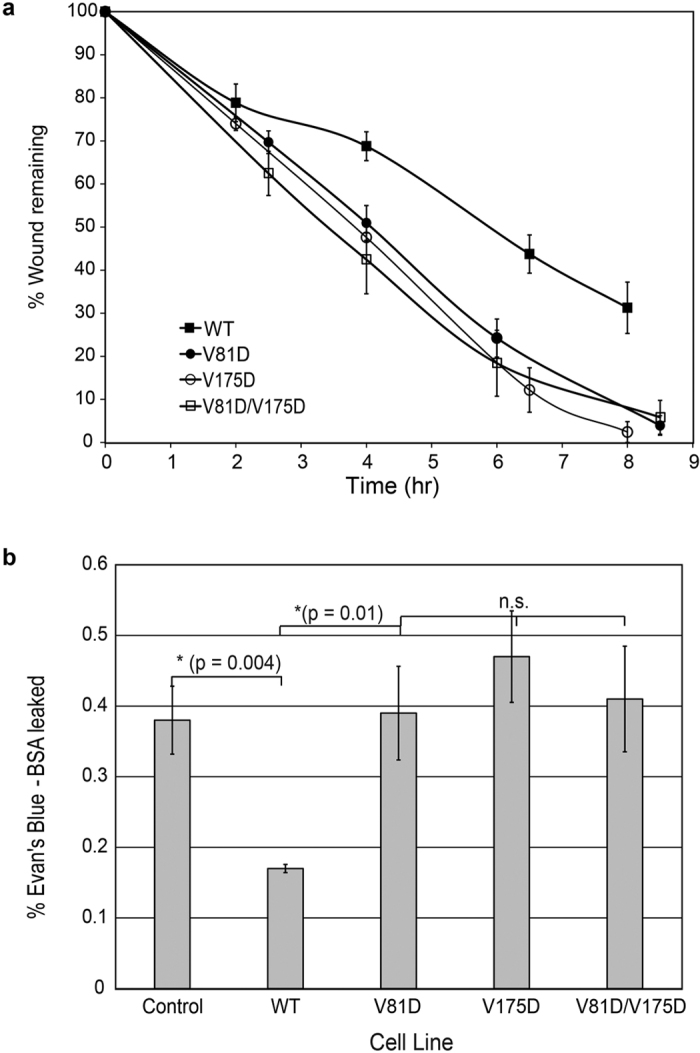
Putative lateral interface mutants impair intercellular barrier integrity and wound healing. **(a)** The percentage of (similar width) wound area remaining as a function of time after scratch wounding a monolayer of A431D cells expressing WT Hu-E-Cad-GFP (black squares) or its mutants V81D (black circles), V175D (white circles) or V81D/V175D (white squares) at similar surface densities. The lines through the data are to guide the eye only. **(b)** Percentage of Evan’s Blue-labeled BSA leaked through cell-cell junctions from the basal chamber, as a function of time. Paracellular permeability measurements were performed with confluent monolayers of A431D cells expressing the different indicated Hu-E-Cad-GFP variants cultured on a transwell support.

We compared the potential physiological impact of the *cis* interface mutants with the altered binding kinetics using two, cell-based assays: scratch wound healing (Fig. 5a) and paracellular permeability (Fig. 5b). In scratch wound healing assays, A431D cells stably expressing the *cis* interface mutants closed wounds much faster than cells expressing WT Hu-E-Cad-GFP (Fig. 5a). Within nine hours, the cells expressing WT Hu-E-cadherin closed only ~70% of the wound, whereas cells expressing the *cis* interface mutants completely closed wounds of similar size, within the same time frame (Fig. 5a). At 4hrs, for example, the remaining wound area was greater for cells expressing WT Hu-E-Cad-GFP relative to the V81D mutant (p = 0.005, N = 6), but all mutants were similar (p > 0.3 for all pairwise comparisons; N = 6 in each case).

We also compared the paracellular permeabilities of confluent A431D cells expressing Hu-E-cadherin-GFP or the *cis* interface mutants. The expression of WT Hu-E-Cad-GFP reduced epithelial barrier permeability significantly, relative to untransfected A431D cell monolayers (Fig. 5b). This result confirmed the formation of interepithelial adherens junctions, in cells expressing WT E-Cadherin^41^. However, the paracellular flux through monolayers of cells expressing the E-Cadherin *cis* interface mutants was statistically similar to the non-transfected control cells (Fig. 5b, p > 0.1). Namely, in the 2.5 hr observation time, less than 0.2% of the dye leaked across monolayers of cells expressing WT E-cadherin. The latter was significantly lower than the flux through control monolayers or monolayers of cells expressing the mutants (control vs WT E-cadherin; p = 0.004, N = 6). The paracellular flux through either control cell monolayers or through monolayers expressing the mutants were statistically similar (p > 0.05 for all pairwise comparisons; N_exp_ = 6, for each condition).

## Discussion

The kinetic signatures of cadherin-mediated intercellular binding reveal enhanced protein-protein interactions within confined adhesion zones. Adhesion frequency measurements of cadherin-mediated intercellular binding exhibit unique, two-stage kinetics described by a rapid initial increase in binding probability to P1 (Fig. 3a), followed by a short lag and then a further, slower increase to a final steady state plateau at P2 (Fig. 3b). The first, fast binding step is due to *trans* (adhesive) dimerization between the N-terminal EC1 domains (Fig. 1b). The cadherin-inactivating W2A mutation abrogated the fast, initial binding step, and point mutations at the *trans*-binding interface quantitatively altered the affinity of the initial binding interaction^38^,^40^. The onset of the second probability increase thus reveals the formation of additional cadherin interactions at the cell-cell junction that were not detected in solution binding assays.

The impact of the *cis* (lateral) binding interface mutants (see Figs 1c and 3a) on the kinetic signatures provided compelling evidence that the second kinetic step is due to lateral ectodomain interactions. All three mutants V81D, L175D and V81D/L175D impeded or blocked the second kinetic process, without altering the initial *trans* binding affinities. These same recombinant cadherins also exhibited similar *trans* binding affinities in solution^13^. The increased binding probability attributed to lateral cadherin association may also be explained by cooperativity between adhesive and lateral interactions predicted by simulations^7^.

The effects of N-glycosylation on the two-stage binding kinetics of neural N-cadherin further support our interpretation of the kinetics signature^34^. De-glycosylating N-cadherin ectodomains—thereby removing possible steric impediments to lateral association—accelerated the rise to P2^34^. However, E-cadherin hypo-glycosylation, which would reduce lateral steric interference, resulted in tighter inter-epithelial junctions^42^. Conversely, removing the EC3 domain near the putative *cis* interface or deleting EC3–5 abolished the second kinetic step^38^. Although EC3 is not directly involved in the putative lateral binding interface, a possible explanation for the latter results is that the EC3 deletion allosterically disrupts the lateral interactions. There is precedence for such long-ranged perturbations. Mutations at the EC2/EC3 junction disrupt *trans* binding, and antibodies against ectodomain epitopes outside the binding site, as well as the phosphorylation state of the cytoplasmic binding partner p120 catenin, alter E-cadherin affinity^33^,^43^,^44^,^45^,^46^,^47^. The kinetic data cannot directly verify the molecular details of the cadherin interactions, and it is currently not possible to determine the oligomeric state of cadherins within the first few seconds of intercellular contact. Nevertheless, the kinetic effects of disrupting putative lateral (*cis*) bonds or de-glycosylating ectodomains support a model in which the two-stage kinetic signature reflects initial *trans* (adhesive) binding, followed by confinement-enhanced lateral interactions that further increase the binding probability.

At the low cadherin surface densities used, the putative oligomerization at the onset of the second kinetic step likely involves only a few proteins. At reported cadherin (and membrane protein) diffusivities from 0.036 down to 0.002 μm^2^/s^48^,^49^,^50^, it could take 7 to 80s, respectively, for two cadherins on the same membrane to collide. The closer proximity of the cadherins bound by bivalent antibodies on the RBCs might accelerate the process. Our results would nevertheless suggest that the ‘cluster’ sizes are small. Future comparisons with kinetic models should address this issue.

The kinetics revealed cadherin interactions that presage the organization of cadherin at cell-cell junctions. Between A431D cells expressing E-cadherin, the rapid rise to P2 corresponded with the formation of large cadherin puncta and tight intercellular barriers, apparent from permeability and wound healing results. Conversely, a slowed (or abolished) rise to P2 indicated impaired cadherin interactions that in turn resulted in smaller, more diffuse cadherin clusters, leakier inter epithelial junctions, and increased wound-healing rates. The *cis* interface mutants V81D and V175D similarly reduced the rate of N-cadherin-dependent spheroid formation by L-cells, compared to cells expressing WT N-cadherin, and the cell boundaries were less defined^51^. N-cadherin mutants postulated to disrupt lateral interactions also reduced the number of cadherin adhesions and the junction lifetimes^52^.

The increase in binding probability after initial adhesive (*trans*) binding, suggests that intermembrane confinement facilitates additional (*cis*) cadherin interactions. Simulations predicted that inter membrane pinning (*trans* bond formation) and consequent reduction in cadherin fluctuations would reduce entropic repulsion between proteins, and enable lateral protein association^8^. Consistent with both this postulate and the kinetic data, lateral interface mutants impeded the assembly of large clusters at cell-cell junctions^26^, but not the formation of small clusters outside of adhesion zones^14^,^26^. Thus, the lateral interface mutants appear to primarily alter the kinetics, after the formation of adhesive bonds—that is, within intercellular gaps. Taken together, these findings support our postulate that the kinetic signature reflects rapid, initial *trans* binding, followed by lateral cadherin interactions, which manifest at intercellular junctions but not in solution.

Other investigations of the potential influence of confinement on lateral cadherin interactions and junction assembly focused on adhesion plaque nucleation. Cluster nucleation involves other, active biochemical processes that could eclipse the biophysical influence of confinement on ectodomain interactions^14^,^27^,^29^,^30^,^53^,^54^,^55^. In this study, the pre-steady-state binding events during the initial 40s of cell-cell contact were independent of the cadherin cytoplasmic domain and cytoskeletal interactions (Table 1); namely, both recombinant ectodomains and membrane bound WT E-Cad exhibited two-stage binding kinetics. The mutants exhibited similar *trans* (adhesion) binding kinetics/affinities, and mutants V81D and L175D exhibited residual putative lateral interactions; consequently, observed cluster nucleation alone may not be sufficient to distinguish subtler differences between the mutants and WT protein.

The effects of mutants on wound healing, for example, were indistinguishable from each other, although all three *cis* mutants altered wound closure rates relative to WT E-Cadherin. The indistinguishable differences in wound healing rates among the mutants may not be surprising because other processes contribute to collective cell migration, and could mask subtler binding differences. For example, cadherins exit junctions through a ‘X-dimer’ intermediate (Fig. 1a)^56^, and this transition state (or other processes) could dominate junction-remodeling kinetics.

Similarly, tight junctions are well-known to regulate barrier permeability^12^. Cadherins are required for tight-junction assembly and maintenance, and disrupting cadherin adhesions contributes to increased barrier leak in several examples^42^,^57^,^58^,^59^. Because of the additional influence of tight junctions, permeability changes may also not be sensitive to more subtle differences between cadherin mutants.

In summary, these pre-steady-state cadherin binding kinetics revealed distinct, early cadherin ectodomain interactions, which contribute to cadherin organization at intercellular junctions and barrier function. Importantly, these kinetic data exposed a distinct, quantifiable kinetic process consistent with confinement-enhanced, lateral cadherin interactions. The identification of a quantitative, biophysical signature will enable future model testing and possible quantification of confinement-dependent perturbations to cadherin assembly at intercellular junctions.

## Additional Information

**How to cite this article**: Shashikanth, N. *et al.* Kinetic Measurements Reveal Enhanced Protein-Protein interactions at Intercellular Junctions. *Sci. Rep.* **6**, 23623; doi: 10.1038/srep23623 (2016).

## Figures and Tables

**Table 1:** Best-fit values of K_2D_ and k_off_ determined from kinetic data.

LHS (cadherins/μm^2^)*	RHS(cadherins/μm^2^)	K_2D_(x 10^−4^ ) μm^2^	k_off_ (sec^−1^)
E-Cad-His_6_ WT (21)	E-Cad-His_6_ WT (21)	3.3 ± 0.5	1.0 ± 0.2
E-Cad-His_6_ L175D (22)	E-Cad-His_6_ L175D (22)	3.0 ± 0.4	0.9 ± 0.1
E-Cad-His_6_ V81D (24)	E-Cad-His_6_ V81D (24)	2.4 ± 0.3	0.9 ± 0.2
E-Cad-His_6_ V81D/L175D (16)	E-Cad-His_6_ V81D/L175D (16)	3.5 ± 0.5	0.9 ± 0.2
A431D Hu-E-Cad WT (7)	E-Cad-His_6_ WT (40)	3.1 ± 0.4	1.2 ± 0.3
A431D Hu-E-Cad V81D/V175D (8)	E-Cad-His_6_ V81D/L175D (40)	2.9 ± 0.3	1.1 ± 0.2

Left Hand Side (LHS) and Right Hand Side (LHS) refer to the cells held by the opposing micropipettes. The right side is the Red Blood Cell labeled with the indicated E-cadherin ectodomain. The cadherin densities (number/μm^2^) in parentheses were quantified by flow cytometry.

^*^If the identity of the cell on the LHS is not given, then a Red Blood Cell labeled with the E-cadherin ectodomains indicated was used, in order to quantify the binding properties of isolated ectodomains.
