# Corrigendum: Kinetic Measurements Reveal Enhanced Protein-Protein Interactions at Intercellular Junctions

**DOI:** 10.1038/srep41469

**Published:** 2017-02-09

**Authors:** Nitesh Shashikanth, Meridith A. Kisting, Deborah E. Leckband

Scientific Reports 6: Article number: 23623doi: 10.1038/srep23623; published online: 03 24 2016; updated: 02 09 2017

This Article contains an error in the Acknowledgments section where:

“This work was supported, in part, by National Institutes of Health Grant 5 RO1 GM097443 (to D.E.L.) and by National Science Foundation (NSF) Grant CMMI 10-29871 (to D.E.L.)”.

Should read:

“This work was supported, in part, by National Institutes of Health Grant 5 RO1 GM097443 (to D.E.L.) and by National Science Foundation (NSF) Grant NSF CBET 11-32116 (to D.E.L.)”.

